# A higher percentage of Higher Education Academy (HEA) qualifications among universities’ staff does *not* appear to be positively associated with higher ratings of student satisfaction. A Letter of Concern in response to Nurunnabi et al MethodsX6 (2019) 788–799

**DOI:** 10.1016/j.mex.2020.100911

**Published:** 2020-05-13

**Authors:** Philip M. Newton, Michael B. Gravenor

**Affiliations:** Swansea University Medical School. Swansea University, SA2 8PP. United Kingdom*.*

We write in response to a recent paper by Nurunnabi *et al* “Does teaching qualification matter in higher education in the UK? An analysis of National Student Survey data”, published Open Access in *MethodsX* Vol 6 p788-799, 2019.

The main finding is stated in the third line of the abstract as

“The findings reveal that a higher percentage of Higher Education Academy (HEA) qualifications among universities’ staff is positively associated with higher ratings of student satisfaction”.

Unfortunately we suggest that the data, as presented, do not support this statement. Furthermore there is evidence to suggest that the data, as included in the paper, suggest no association between HEA qualifications and ratings of student satisfaction.

If the aforementioned statement by Nurunnabi *et al* were correct, it would represent an important finding for the UK Higher Education (HE) sector and would contradict existing published findings [1]. Any outcome from the analysis conducted by Nurunnabi et al is important, *as long as the analysis is correct.* Student satisfaction, particularly as measured using the National Student Survey (NSS) data analysed by Nurunnabi *et al*, is a very powerful metric in UK Higher Education (HE). Due to clustering and ceiling effects in NSS data, small differences in overall satisfaction can send a university up, or down, many places in league tables and thus affect student recruitment in ways that can potentially cost, or benefit, universities by significant sums of money. Universities are therefore highly motivated to identify factors associated with student satisfaction.

Many UK universities have invested heavily in schemes to develop the ‘HEA Qualification’ as identified by Nurunnabi *et al.* Fellowship of the Higher Education Academy (HEA, now called ‘AdvanceHE’) is achieved by submission of a fee, and a reflective document which demonstrates compliance with the UKPSF (the accreditation standards for HEA Fellowship). Much of the investment is in the institutional accreditation of schemes to develop Fellows, wherein staff are employed by universities to oversee the Fellowship process. Accredited Postgraduate Certificates of Education may also be undertaken by university staff in order to achieve Fellowship. The cost of these endeavours is significant, particularly when including opportunity costs such as the staff time required to undertake Fellowship or a Postgraduate Certificate, and the consequent time away from revenue-earning activities such as teaching, research and enterprise.

Thus it is of significant importance to the UK HE sector to have accurate analyses of whether HEA Fellowship is associated with student satisfaction. For this reason we feel compelled to write and identify our concerns with the analysis undertaken by Nurunnabi *et al*, and to request that the paper be clarified, corrected or retracted. If a subsequent clarification reveals that our concerns are unfounded and then original conclusion stands, then we will withdraw this Letter of Concern.

The concerns are listed below. We have attempted to rank these according to what we believe to be their significance, although this is not always possible as there are some aspects which we do not have the full data for.

**Fig. 1:**
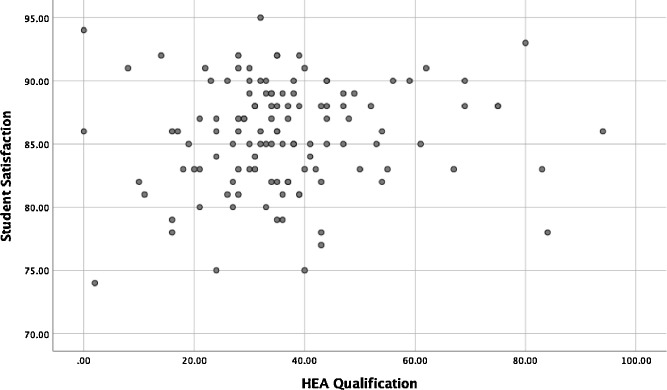
Scatter Plot of HEA Qualification and Student Satisfaction data from Table 1 of [2].

1. A simple Pearson test for HEA teaching qualification against NSS score (columns 2 and 3 of table 1) does not return a significant correlation (P=0.324). It appears that the authors have also run this test, as all the values for ‘r’ are shown in Table 5 (we get exactly the same value), yet have appeared to ignore the implications as this value would appear to cast considerable doubt on the main finding identified above.
2. Figure 9 is purported to show, according to the text on p795 *“that the HEA Teaching Qualification is associated with higher student satisfaction”.* Again, we can reproduce this figure exactly (from columns 2+3). There does not appear to be a significant association, and a regression of the two variables returns a non-significant trend (p = 0.32, as would be expected from the correlation coefficient above), and HEA_Qual explains less than 1% of the variation in NSS score.
3. Table 6 appears to be where the authors derive their statistical conclusion that there is a relationship between HEA qualifications and NSS scores. Again, we can repeat the analysis. In this case a precise replication is not possible because the data “Total_staff” were not provided. We based our replication on the total academic staff per HE institution (excluding atypical contracts) available from the HESA records for the academic year 2014-15 (to correspond with the NSS scores which were from 2015). There are several claims made in the paper regarding the regression model:
  a. "It is evident from the results that model fits the data well (p < .05)". We cannot replicate this finding. In contrast we find that overall fit of the model with 3 degrees of freedom is not significant (p = 0.13). The model does not fit the data well.
  b. "*and there is a strong positive relationship between dependent variables and independent variables*"*.* We cannot replicate this finding. In contrast, we find no significant relationship between any of the independent variables and Student_Satisf. This reiterates the (non-significant) correlations noted above, and is also evident from basic scatter plot of the data (see Fig. 1, 2 below).

**Fig. 2:**
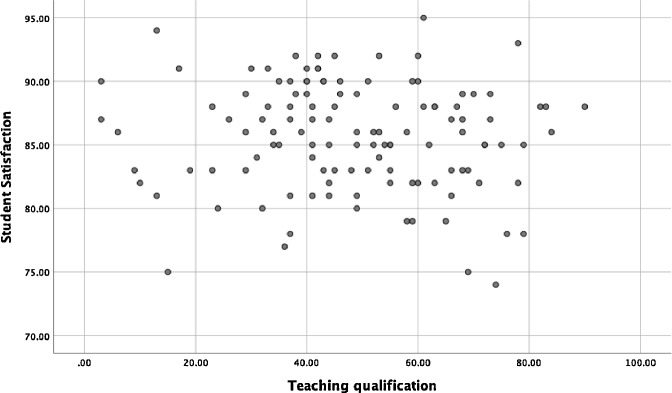
Scatter Plot of Teaching Qualification and Student Satisfaction data from Table 1 of [2].
  c. "*The independent variables of the model explain 34% of the variations in the dependent variable*"*.* We cannot replicate this finding. In contrast we find the R2 for the full model to be almost an order of magnitude less, at only 4.8%. Even if different Total_Staff sources are used, these estimates are difficult to reconcile, and we note our estimate is more consistent with all other statistics and graphs reported in the actual paper (and reproduced here), such as the lack of correlation. We also note that most of this (small) variation is explained by Total_staff. The combined effect of HEA_Qual and Teaching_Qual is only 2% of NSS variance.
  d. "*The variables when compared on individual basis, only faculties with HEA qualification variable is significant (p < .05)*"*.* We cannot replicate this finding. There are no significant variables in the model.
  e. "*This reveals that faculties with HEA qualification in universities is positively associated with student satisfaction*". We cannot replicate this finding. In contrast we find the opposite, as is supported by most analyses supplied by actual paper. All analyses: univariable, multivariable regression, and simple scatter plots, contradict this claim.
4. The legend for Table 6 states that ‘the bold value indicates p<.05’. There is only one bold value in the table, and it is 0.067 (i.e. greater than 0.05).
5. The authors show good practice to include the raw data in the paper. However, we believe it is not clear in the manuscript as currently presented, what the first two of these columns mean (in table 1). The text, under ‘*Sample’* on p790, appears to indicate that column one is “the percentage of faculty with total teaching qualification (including HEA qualification and other teaching qualifications)” but then in the table the numbers in that column are often lower than the next column (2), which is HEA qualifications only. Thus the numbers in column one should be higher than, or equal to, those in column 2? We understand, from a response from the authors, that column 2 refers to the percentage of teaching qualifications which are HEA qualifications. We believe it is important to clarify this in the paper. We have used the information presented in columns 1 and 2 to calculate a revised version of column 2, which shows the percentage of total staff who hold HEA Fellowship. We have then run a Pearson test on this against the NSS scores (column 3) and still do not find a remotely statistically significant association (r = 0.061, n=121, p=0.509)
6. The data analysed are from 2015, with some from 2014. Any findings should be tempered with emphasis that they arise from only one year of data, when considerably more are available.
7. On p798 the authors state that *“Pearson Correlation shows that there was a positive correlation between HEA Qualification and Teaching Qualification, which was statistically significant (r=0.242, n=121, p=.008). This means that most of the qualified teachers were HEA qualified”*. We do not believe the analysis supports the conclusion as stated in the second sentence. If, at every HEI, 1% of the qualified teachers were FHEA, there would be a strong correlation but a minority of the qualified teachers would be FHEA. As it is, the average of column 2 is 37%, suggesting that it is in fact a minority, although this is difficult to conclude firmly without presenting and analyzing the total numbers of staff at each institution
8. The analysis and text makes repeated reference to distinctions between ‘Russell Group’ and ‘non Russell Group’ universities. This is not ever justified or explained. It is not clear to us why this distinction is important for the main research questions and analysis?
9. The abstract states that ‘121 universities were randomly selected’ but the means of randomly selecting those universities is never explained. It appears that the authors have selected almost all universities in England, and have excluded Wales, Scotland and Northern Ireland.
10. The analysis on p795 and in Fig. 2 claims that there are difference in student satisfaction between England, Wales, Northern Ireland and Scotland. However the data from the three other countries (i.e. not England) are never presented (i.e. in Table 1) and no actual analysis is presented to determine the significance (or otherwise) of these differences.

These are just some of the issues we have identified. We are most concerned about points 1-4. We would be grateful if these could be addressed. We are happy to provide any clarification if it would help.

**Sincerely**

Professor Philip M. Newton

Professor Michael B. Gravenor

**Swansea University Medical School**
